# SENP1-mediated deSUMOylation of USP28 regulated HIF-1α accumulation and activation during hypoxia response

**DOI:** 10.1186/s12935-018-0722-9

**Published:** 2019-01-03

**Authors:** Shi-chun Du, Lan Zhu, Yu-xing Wang, Jie Liu, Die Zhang, Yu-lu Chen, Qing Peng, Wei Liu, Bin Liu

**Affiliations:** 1 Department of Endocrinology, Xin Hua Hospital Affiliated to Shanghai Jiaotong University School of Medicine, Shanghai, 200092 People’s Republic of China; 2 School of Molecular Sciences and Biodesign Center for Applied Structural Discovery, Biodesign Institute, Arizona State University, Tempe, AZ 85287 USA; 3 Key Laboratory of Protein Modification and Tumor, Hubei Polytechnic University School of Medicine, Huangshi, Hubei 435003 People’s Republic of China; 4 Department of Pulmonary Medicine, Minhang Hospital, Zhongshan Hospital, Fudan University, Shanghai, 201199 People’s Republic of China

**Keywords:** USP28, HIF-1α, SENP1, Hypoxia

## Abstract

**Background:**

The ubiquitin-specific protease 28 (USP28) is an oncogenic deubiquitinase, which plays a critical role in tumorigenesis via antagonizing the ubiquitination and degradation of tumor suppressor protein FBXW7-mediated oncogenic substrates. USP28 controls hypoxia-dependent angiogenesis and metastasis by preventing FBXW7-dependent hypoxia-inducible transcription factor-1α (HIF-1α) degradation during hypoxia. However, it remains unclear how USP28 activation and HIF-1α signaling are coordinated in response to hypoxia.

**Methods:**

The in vitro deubiquitinating activity assay was used to determine the regulation of USP28 by hypoxia. The co-immunoprecipitation and GST Pull-down assays were used to determine the interaction between USP28 and SENP1. The in vivo deSUMOylation assay was performed to determine the regulation of USP28 by SENP1. The luciferase reporter assay was used to determine the transcriptional activity of HIF-1α.

**Results:**

Here, we report that USP28 is a SUMOylated protein in normoxia with moderate deubiquitinating activity towards HIF-1α in vitro, while hypoxia and HIF-1α activate USP28 through SENP1-mediated USP28 deSUMOylation to further accumulate HIF-1α protein in cells. In agreement with this, a SUMOylation mutant USP28 showed enhanced ability to increase HIF-1α level as well as control the transcriptional activity of HIF-1α.

**Conclusion:**

Collectively, our results reveal a novel SENP1–USP28–HIF-1α positive feedback loop to maximize the concentration of HIF-1a protein and amplify its downstream effects during hypoxia response.

## Background

The hypoxia-inducible transcription factor-1α (HIF-1α) has central roles in angiogenesis, carcinogenesis, and cell adaptions to hypoxic conditions both transcriptionally dependent and independent [1, 2]. Thus, the protein levels of HIF-1α must be tightly regulated to prevent its inappropriate activation. Indeed, the E3 ubiquitin ligase, von Hippel–Lindau (VHL) mediates the ubiquitination and degradation of HIF-1α in normoxia condition [3]. It has been demonstrated that HIF-1α is also degraded by glycogen synthase kinase-3 (GSK-3) and FBXW7-mediated ubiquitination, and could be antagonized by ubiquitin-specific protease 28 (USP28) in hypoxia condition [4, 5]. However, it is well-known that the HIF-1α protein was accumulated with increased protein stability during hypoxia, suggesting that either the elements that promoted HIF-1α degradation were repressed or that stabilized HIF-1α were enhanced.

SUMOylation is a highly dynamic process that could be reversed by SUMO1/sentrin specific peptidase (SENP) family members and appears to be dysregulated in diverse cancer types [6, 7]. HIF-1α is modified through different pathways, such as acetylation, phosphorylation, ubiquitination as well as SUMOylation during hypoxia [8–10]. Interestingly, as a transcriptional factor, HIF-1α could induce the expression of SENP1, which in turn deSUMOylates HIF1a, therefore forming a positive feedback loop to stabilize HIF-1α during hypoxia [11, 12]. USP28 has been also identified as a SUMOylation substrate, and SUMOylation at its N-terminal domain inhibits its deubiquitinating activity [13]. Thus, it could be critical to investigate whether USP28 was involved in the HIF-1α positive feedback loop during hypoxia.

The aim of this study is to clarify the relationship between USP28 and HIF-1α during hypoxia. We found that USP28 is SUMOylated in normoxia but rapidly deSUMOylated under hypoxia condition. SENP1-mediated deSUMOylation of USP28 enhanced its deubiquitinating activity towards HIF-1α, and thereby prevented FBXW7-dependent degradation of HIF-1α during hypoxia. Our results indicate a novel mechanism by which SENP1-mediated deSUMOylation of USP28 stabilized HIF-1α during hypoxia.

## Materials and methods

### Cell culture

HCT116, A549 and 293T cells were obtained from The Cell Bank of Type Culture Collection of Chinese Academy of Sciences (CAS, Shanghai, China), and cultured in DMEM supplemented with 10% heat-inactivated fetal bovine serum (FBS, Hyclone, Logan, UT, USA), 100 IU/ml penicillin and 100 mg/ml streptomycin, maintained at 37 °C in a humidified atmosphere with 5% CO_2_.

### Plasmids and stable cell line construction

Wild type USP28 construct (#41948) was obtained from addgene and was used for subcloning into pcDNA-Flag vector. USP28KR was created with site-directed mutagenesis (Stratagene). Wild type HIF-1a construct (#21101) was obtained from addgene and was used for subcloning into pcDNA-HA vector. SUMO2 and SENP1 were amplified from 293T cells by PCR and cloned into the pbabe- or pEGFP-vector, respectively. The stable cell line construction has been described previously. For brief, viral supernatants were produced in HEK293T cells co-transfected with the pBabe-control or pBabe-his-SUMO2 constructs and packaging vectors. Viral supernatants were collected 48 h after transfection, filter-sterilized, and added to the HCT116 cells with 10 μg/ml Polybrane for 48 h and selected with puromycin (1 μg/ml) for 3 days.

### Western blot

Cells were harvested and lysed with cold lysis buffer (150 mM Tris–HCl, pH 6.8, 100 mM DTT, 2% SDS and 10% glycerol). Proteins were separated by 10–12% SDS-PAGE, transferred to NC membranes. Western blot assay was then performed by using the following antibodies: anti-USP28, anti-SENP1, anti-GST, anti-His, anti-GFP, anti-GAPDH (Santa Cruz, CA, USA), anti-HIF1a (H1alpha67, abcam), anti-Myc (Cell Signaling, Boston, MA, USA), anti-Flag M2 and anti-HA (Sigma, St Louis, MI, USA).

### Immunoprecipitation

The immunoprecipitation process has been described previously with some modifications [14, 15]. Briefly, cells transfected with Flag-USP28 and GFP-SENP1 were lysed in cold NETN buffer (100 mM NaCl, 1 mM EDTA, 20 mM Tris–HCl, pH 8.0, 0.5% NP-40) for 45 min at 4 °C. Cell lysates were centrifuged, and the supernatants were incubated with M2 beads for 2 h at 4 °C. The beads were washed with NETN buffer, and the bound proteins were eluted with Flag peptide and subjected to western blot with indicated antibodies.

### SUMOylation and Ubiquitination assays

HCT116 cells were co-transfected with his-SUMO2 or his-Ubiquitin and other indicated plasmids for 36 h. Cells were sonicated in lysis buffer containing 8 M urea and 10 mM imidazole. His-SUMO2 or his-Ubiquitin conjugated proteins were recovered with Ni–NTA resin (Qiagen), washed with urea lysis buffer containing 20 mM imidazole, and eluted with buffer containing 5% SDS, 0.72 M DTT, and 200 mM imidazole. Proteins were separated and visualized by western blot assay.

### GST pull-down

The whole procedure of GST pull-down has been described previously [16]. Briefly, the purified GST or GST-SENP1 proteins were incubated with the purified USP28 protein for 1 h at 30 °C. Then the precipitations were eluted by 2Χ SDS loading buffer and followed by western blot with previously described antibody.

### Luciferase assay

For luciferase reporter assays, cells were seeded in 24-well plates and transfected with HA-HIF-1α, pGL3-HRE-Luc, pSV40-renilla and USP28 plasmids. Cells were then harvested 36 h after transfection. Luciferase activities were measured and assessed using the Dual Luciferase Reporter Assay System (Promega, USA).

### Statistical analysis

Data were shown as mean ± standard deviation (SD). Differences between groups were analyzed using Student’s t-test, and *P* < 0.05 was considered statistically significant. Statistical significance is displayed as *(*P *< 0.05), **(*P* < 0.01) or ***(*P* < 0.001).

## Results

### Hypoxia enhanced the deubiquitinating activity of USP28 on HIF-1α

**Fig. 1:**
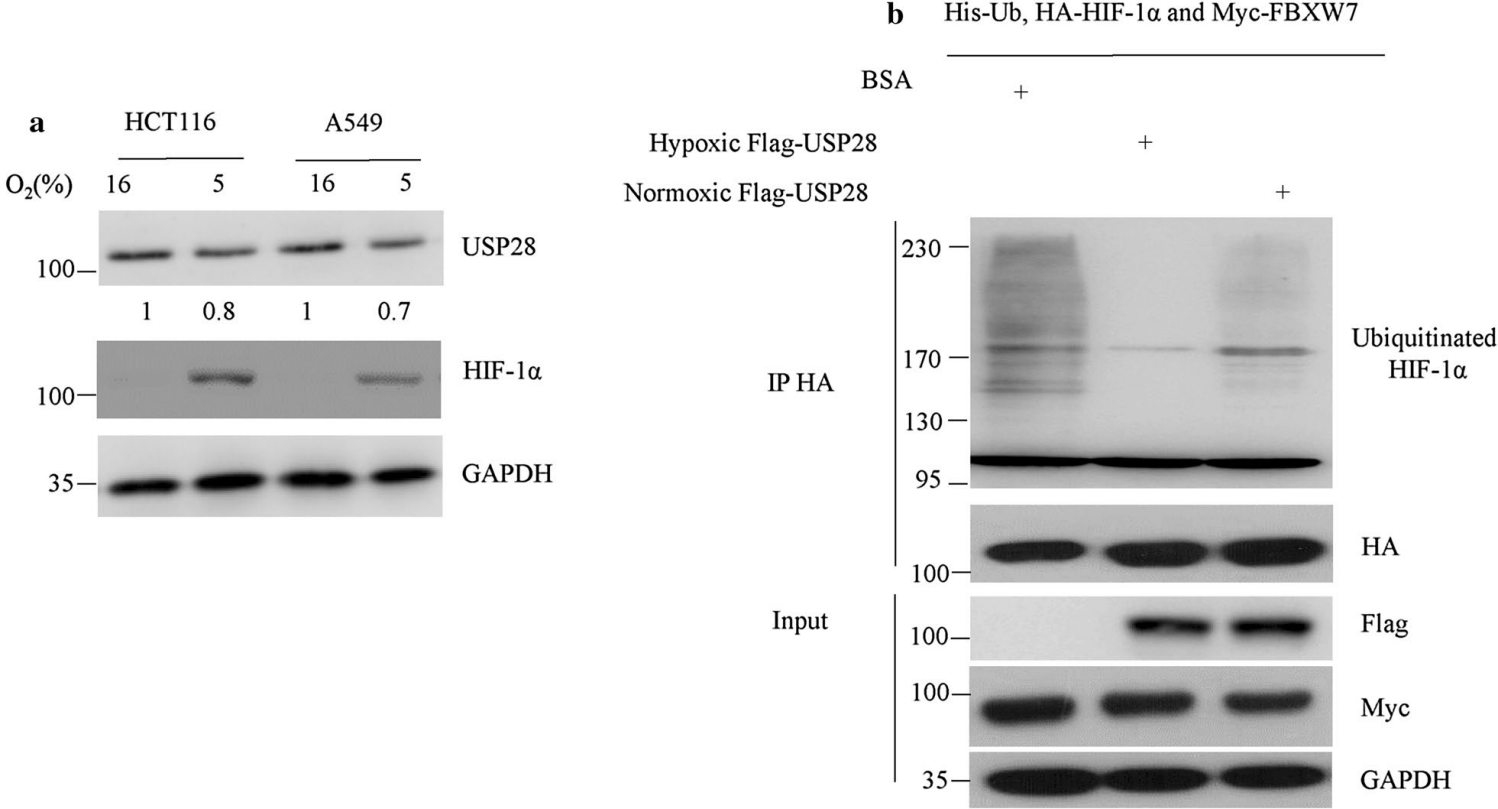
Hypoxia enhanced the deubiquitinating activity of USP28 on HIF-1α. **a** HCT116 and A549 cells were cultured under normoxic (16% O_2_) or hypoxic (5% O_2_) conditions for 24 h. Cells were then lysed and USP28 protein levels were analyzed by western blot. The number on the bottom indicates signal intensity of USP28 protein against GAPDH. **b** 293T cells were transfected with Flag-tagged USP28 for 24 h and then cultured under normoxic or hypoxic conditions for additional 24 h. Then, Flag M2 beads were used to purify USP28 proteins in 293T cells. HA-HIF-1α, Myc-FBXW7 and His-Ubiquitin were co-transfected in 293T cells for 36 h. MG132 was added 4 h before cells were harvested. Ubiquitinated HIF-1α was purified from 293T cells using anti-HA antibody and used as the substrate of USP28 in vitro. BSA, normoxic USP28 or hypoxic USP28 proteins were added to ubiquitinated HIF-1α-FBXW7 complex for 1 h at 30 °C. 2Χ SDS loading buffer was then added to terminate the enzymatic reaction and the materials were subjected to western blot with indicated antibodies

It has been reported that USP28 was down-regulated by hypoxia at both mRNA and protein levels [4]. However, the decline of USP28 cannot elucidate why USP28 is able to antagonize FBXW7-mediated HIF-1α degradation during hypoxia, as HIF-1α protein was dramatically accumulated in hypoxia. Thus, we reexamined these experiments using both HCT116 and A549 cells. The results display a slightly (about 20%) decrease of USP28 protein during hypoxia (5% O_2_) in both cell lines (Fig. 1a), suggesting there may be other mechanisms controlling USP28 to prevent HIF-1α from degradation. As the deubiquitinating activity of USP28 is critical for its protection role in HIF-1α degradation, therefore it was closely monitored under hypoxia condition. To this end, 293T cells were transfected with Flag-tagged USP28 for 24 h and then under normoxic (16% O_2_) or hypoxic (5% O_2_) conditions for additional 24 h. Then, Flag M2 beads were used to purify USP28 proteins in 293T cells from both conditions and their activity were compared against ubiquitinated HIF-1α protein. HA-HIF-1α, Myc-FBXW7 and His-Ubiquitin were co-transfected in 293T cells for 36 h. MG132 was added 4 h before cells were harvested. Ubiquitinated HIF-1α was purified from 293T cells using anti-HA antibody and used as the substrate of USP28 in vitro. We found that USP28 purified from hypoxia treated 293T cells showed higher deubiquitinating activity against ubiquitinated HIF-1α comparing to the same protein from normoxia treated cells (Fig. 1b), indicating that hypoxia increased the activity of USP28 on HIF-1α protein.

### Hypoxia and HIF-1α promoted USP28 deSUMOylation

**Fig. 2:**
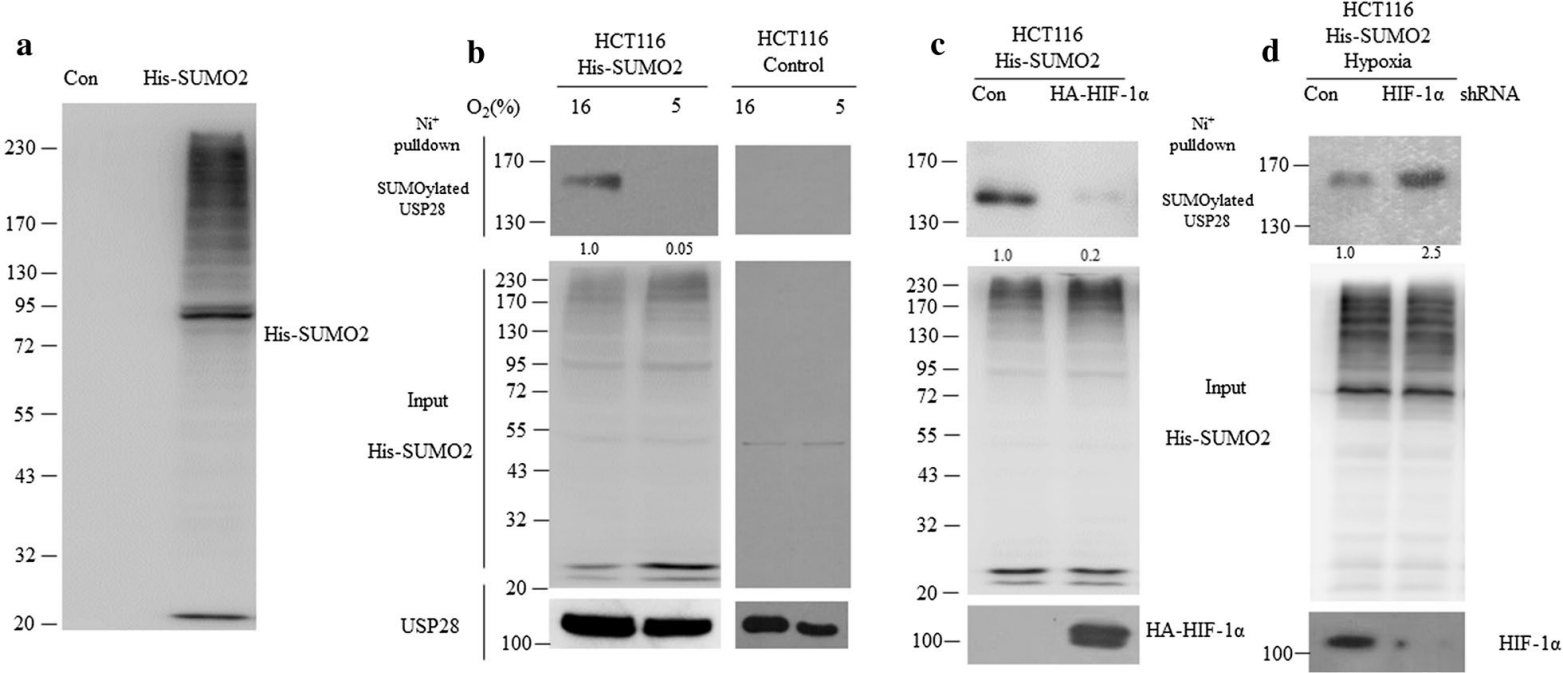
Hypoxia and HIF-1α promoted USP28 deSUMOylation. **a** HCT116 cells stable expressing of His-SUMO2 or vector control was lysed and subjected to western blot with indicated antibodies. **b** HCT116 cells stable expressing of His-SUMO2 and HCT116 cells were cultured under normoxic (16% O_2_) or hypoxic (5% O_2_) conditions for 24 h. SUMOylated proteins were pulled down with Ni^+^ beads. The input and SUMOylated proteins were then subjected to western blot with indicated antibodies. **c** HCT116 cells stable expressing of His-SUMO2 were transfected with vector control or HA-HIF-1α for 36 h, cells were lysed and SUMOylated proteins were pulled down with Ni+ beads and subjected to western blot with indicated antibodies. **d** HCT116 cells stable expressing of His-SUMO2 were transfected with control shRNA plasmid or shRNA against HIF-1α for 24 h and under hypoxic (5% O_2_) conditions for additional 24 h. Cells were harvested and SUMOylated proteins were pulled down with Ni+ beads and subjected to western blot with indicated antibodies

SUMOylation plays a negative role in controlling the deubiquitinating activity of USP28 in vivo [13]. Our data display that hypoxia modulates the activity of USP28, which leads to the question whether hypoxia also regulates USP28 SUMOylation. To shed lights on this mystery, we generated HCT116 cells which stably express His-SUMO2 (Fig. 2a), which were incubated under normoxia or hypoxia conditions for 24 h. HCT116 cells and His-SUMO2 HCT116 cells were harvested and SUMOylated proteins were then pulled down with Ni^+^ beads. We found that the SUMOylation form of USP28 almost disappeared in hypoxia treated cells (Fig. 2b), whereas the global SUMOylation level remained unchanged. It is well known that HIF-1α is the master regulator in hypoxia response, therefore HIF-1α could potentially regulate USP28 deSUMOylation. His-SUMO2 HCT116 cells were transfected with HA-HIF-1α plasmid or HA-vector control plasmid for 36 h. Cells were harvested, and SUMOylated proteins were pulled down with Ni+ beads. As expected, USP28 purified from His-SUMO2 HCT116 cells transfected with HIF-1α plasmid showed decreased SUMOylation level compared with the one from cells with vector transfection (Fig. 2c). Moreover, His-SUMO2 HCT116 cells were transfected with control shRNA plasmid or shRNA against HIF-1α for 24 h and under hypoxic (5% O_2_) conditions for additional 24 h. Cells were harvested and SUMOylated proteins were pulled down with Ni+ beads. We found that without HIF-1α, the SUMOylation of USP28 was increased (Fig. 2d). Taken together, these data suggested that hypoxia and HIF-1α promoted USP28 deSUMOylation.

### SUMOylation of USP28 impaired its deubiquitinating activity on HIF-1α

**Fig. 3:**
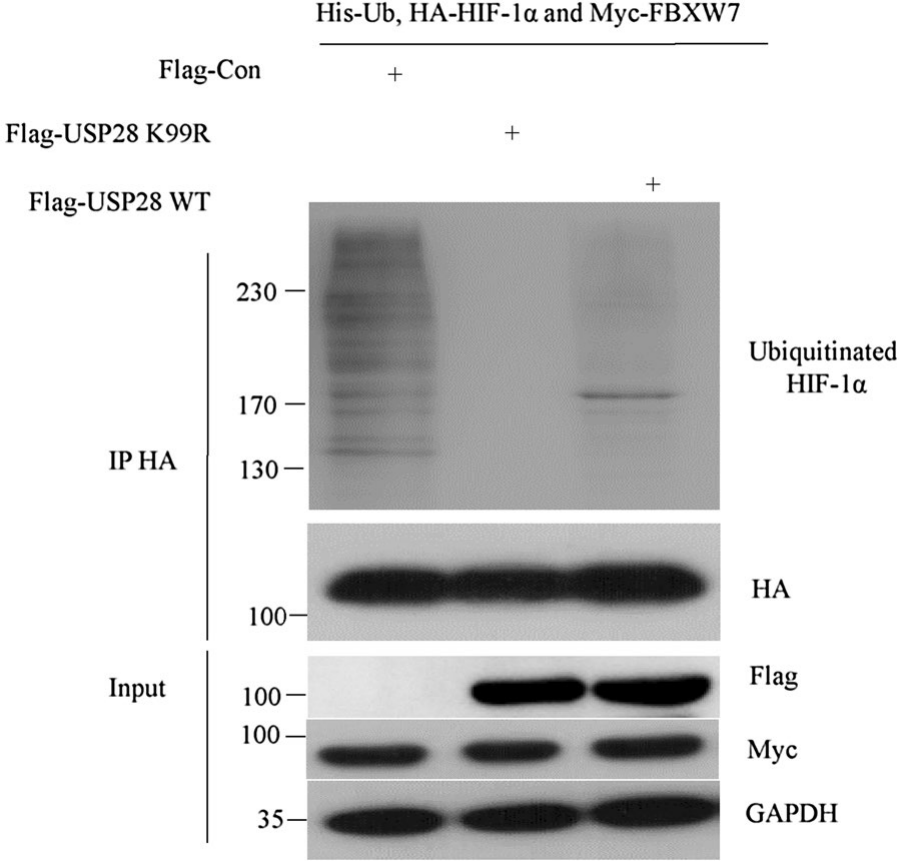
SUMOylation of USP28 impaired its deubiquitinating activity on HIF-1α. 293T cells were co-transfected with Flag-USP28 WT or Flag-USP28 K99R, His-ubiquitin, FBXW7 and HA-HIF-1α for 36 h and MG132 was added 4 h before cells were harvested. Ubiquitinated HIF-1α was purified from 293T cells by using anti-HA antibody and detected by western blot with anti-ubiquitin antibody

It has been demonstrated that K99 is the major SUMOylation site on USP28 that controls its deubiquitinating activity [13]. To investigate the impact of USP28 SUMOylation on HIF-1α, we generated Flag-USP28 K99R mutant plasmid. Flag-USP28 WT or Flag-USP28 K99R was co-transfected with His-ubiquitin, FBXW7, and HA-HIF-1α into 293T cells for 36 h, and MG132 was added 4 h before cells were harvested. Ubiquitinated HIF-1α was purified from 293T cells using anti-HA antibody and detected by western blot with anti-ubiquitin antibody. The K99R mutant USP28 showed much stronger efficiency against HIF-1α ubiquitination in vivo compared with USP28 WT (Fig. 3), suggesting that SUMOylation of USP28 at K99 inhibits its deubiquitinating activity on HIF-1α.

### SENP1 directly interacts with USP28

**Fig. 4:**
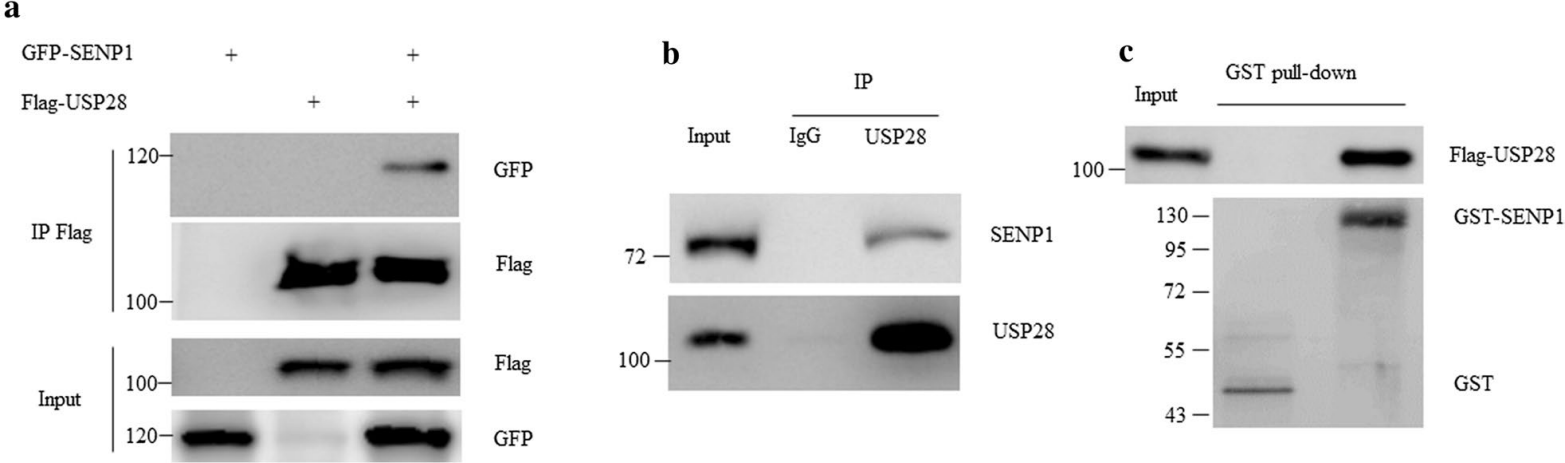
SENP1 interacted with USP28 both in vivo and in vitro. **a** 293T cells were co-transfected with Flag-USP28 and GFP-SENP1 plasmids for 36 h. Cells were harvested and immunoprecipitation was performed with Flag M2 beads. The input and immunoprecipitates were subjected to western blot with indicated antibodies. **b** HCT116 cells were lysed and subjected to immunoprecipitation with mouse IgG or anti-USP28 antibody. The input and immunoprecipitates were subjected to western blot with indicated antibodies. **c** Bacterial purified GST-SENP1 proteins or GST alone were incubated with the purified Flag-USP28 protein for 1 h at 30 °C. Then the reaction was terminated by 2Χ SDS loading buffer and followed by western blot with indicated antibody

Due to lack of the deSUMOylation activity by HIF-1α, we hypothesized that hypoxia and HIF-1α decrease USP28 SUMOylation through a deSUMOylation enzyme. It is well-established that SENP1 serves as a critical substrate of HIF-1α, which harbored hypoxia response elements (HRE) on its promoters and was rapidly accumulated in response to hypoxia [11, 12]. This raised the question whether SENP1 was the enzyme responsible for USP28 deSUMOylation. Firstly, we tested whether SENP1 could interact with USP28. Flag-USP28 and GFP-SENP1 plasmids were co-transfected into 293T cells for 36 h. Cells were harvested, and the immunoprecipitation of Flag-USP28 revealed the presence of GFP-SENP1 protein (Fig. 4a), and SENP1 was also present in immunoprecipitates of endogenous USP28 in HCT116 cell lysate (Fig. 4b). In addition, GST pull-down assay was performed to further study if the interaction between USP28 and SENP1 was direct. We found that bacterial purified GST-SENP1 protein was able to capture the Flag-USP28 protein purified from 293T cells. These data indicate that the deSUMOylation enzyme SENP1 directly interacted with USP28.

### SENP1 deSUMOylated and enhanced USP28 deubiquitinating activity towards HIF-1α

**Fig. 5:**
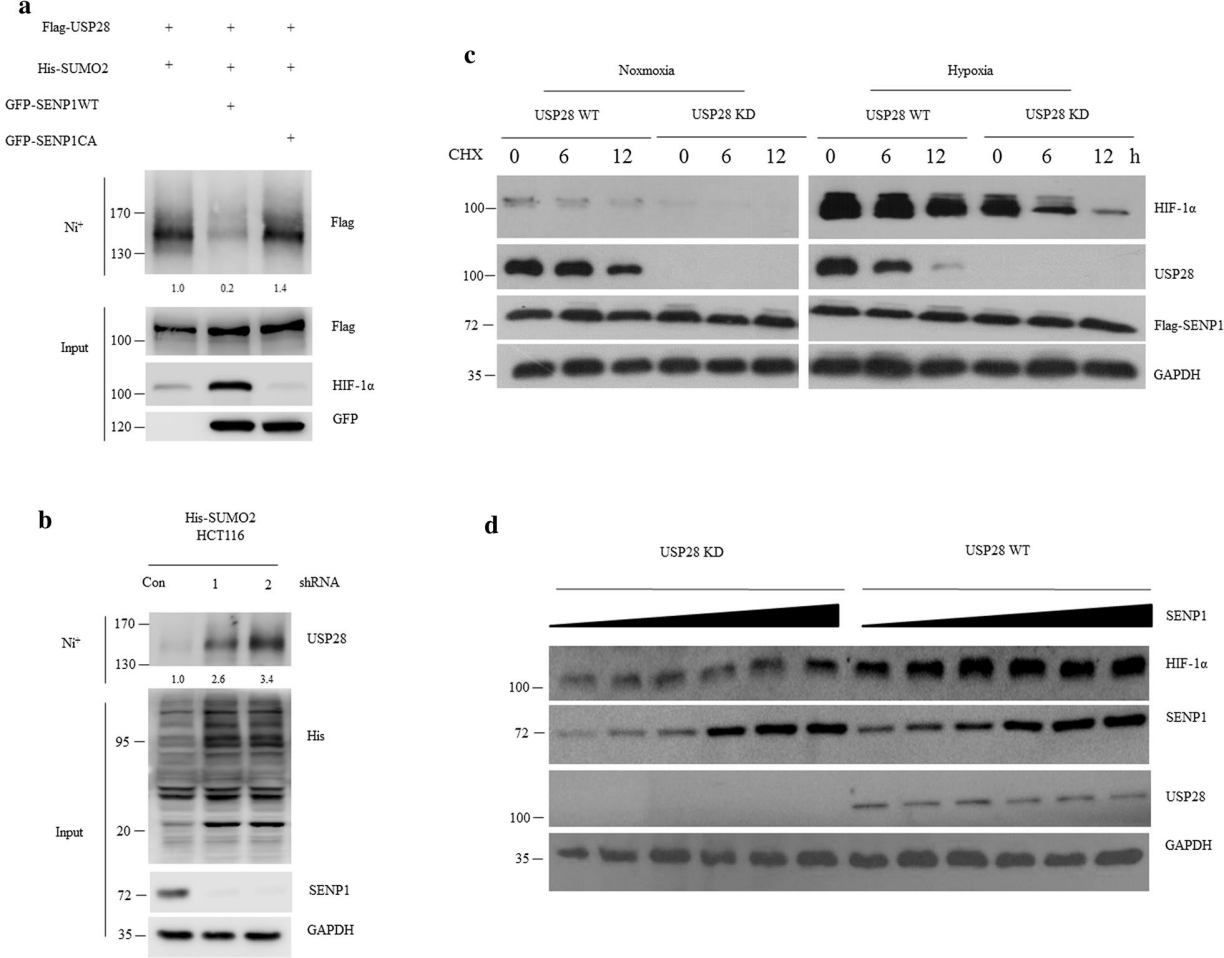
SENP1 deSUMOylated and enhanced USP28 deubiquitinating activity towards HIF-1α. **a** 293T cells were co-transfected with Flag-USP28, His-SUMO2, GFP-SENP1 WT or SENP1 SENP1 CA. SUMO2-conjugated proteins were pulled down by Ni^+^ beads under denaturing conditions and SUMOylated USP28 protein was detected by anti-Flag antibody. **b** His-SUMO2 HCT116 cells were transfected with control or two shRNAs against SENP1, SUMO2-conjugated proteins were pulled down by Ni^+^ beads under denaturing conditions and subjected to western blot with indicated antibody. **c** USP28 KD and USP28 WT HCT116 cells were generated by stable expressing of shRNAs against either USP28 or none specific target. These cells were under either normoxia or hypoxia conditions and treated with 20 μM cycloheximide (CHX) for the indicated timepoints. Cells were then lysed and subjected to western blot with indicated antibodies. **d** USP28 KD or Wild type HCT116 cells were transfected with increased amount of SENP1 plasmid for 36 h, cells were then lysed and subjected to western blot with indicated antibodies

The explicit interactions between SENP1 and USP28 prompted us to further examine whether SENP1 could be a deSUMOylation enzyme for USP28 SUMOylation. To this end, an in vivo deSUMOylation assay was then performed. 293T cells were co-transfected with Flag-USP28, His-SUMO2, GFP-SENP1 WT or SENP1 catalytic mutant (SENP1 CA). SUMO2-conjugated exogenous USP28 was pulled down by Ni^+^ beads under denaturing conditions, and SUMOylated USP28 protein was detected by anti-Flag antibody. Our results display that SENP1 WT removed the SUMOylated USP28 band, but not with SENP1 CA (Fig. 5a). We also silenced the expression of SENP1 in His-SUMO2 HCT116 cells by two pairs of small hairpin RNA (shRNAs), and the knockdown efficiency was confirmed by western blot. Meanwhile, SENP1 KD HCT116 cells showed increased SUMOylated USP28 when compared with SENP1 WT cells (Fig. 5b), and overexpression of SENP1 WT, but not SENP1 CA, further increased HIF-1α abundance in the presence of USP28 (Fig. 5a). We also generated USP28 KD HCT116 cells by stable expressing shRNA against USP28, and compared with USP28 WT cells to measure the half-life of endogenous HIF-1α by cycloheximide (CHX) assay under both normoxia and hypoxia conditions. We found that the HIF-1α proteins were less stable in the absent of USP28 in both cases (Fig. 5c). In addition, silencing of USP28 also largely prevented SENP1-induced HIF-1α stabilization during hypoxia (Fig. 5d). Taken together, these data suggested that SENP1-mediated USP28 deSUMOylation is critical for hypoxia-induced USP28 activation and HIF-1α stabilization.

### Expression of USP28 K99R induces an increase in HIF-1α protein and HIF-1α transcriptional activation

**Fig. 6:**
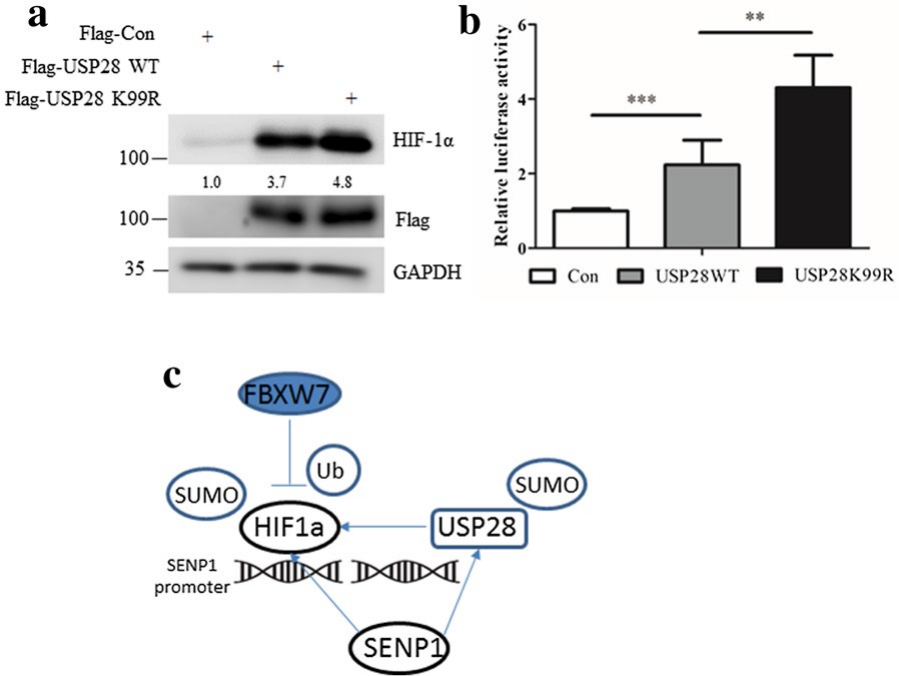
Expression of USP28 K99R induces an increase in HIF-1α protein and HIF-1α transcriptional activation. **a** HCT116 cells were transfected with Flag-tagged vector control, Flag-tagged USP28 WT or Flag-tagged USP28 K99R for 36 h, cells were then lysed and subjected to western blot with indicated antibody. **b** 293T cells were co-transfected with HA-HIF-1α, pGL3-HRE-Luc, pSV40-renilla and Flag-tagged vector control, Flag-tagged USP28 WT or Flag-tagged USP28 K99R for 36 h, after which the luciferase activities in each group were measured and compared, respectively. **c** Proposed model for the SENP1–USP28–HIF-1α positive feedback loop. HIF-1α is able to induce the expression of SENP1, which in turn deSUMOylates and stabilizes HIF1a. We show SENP1 also deSUMOylates USP28 to enhance its deubiquitinating activity towards HIF-1α, and thereby preventing FBXW7-dependent degradation of HIF-1α during hypoxia

To investigate the significance of USP28 deSUMOylation, we analyzed the protein levels of HIF-1α in cells expressing either Flag-tagged vector control, Flag-tagged USP28 WT or Flag-tagged USP28 K99R. USP28 WT slightly increased the expression of HIF-1α, whereas USP28 K99R showed higher HIF-1α protein level, consistent with their SUMOylation status (Fig. 6a). 293T cells were co-transfected with HIF-1α, HRE-driven luciferase with renilla as an internal control, and Flag-tagged vector control, Flag-tagged USP28 WT or Flag-tagged USP28 K99R. Our co-transfection data found that the highest HIF-1α luciferase activity was achieved in USP28 K99R transfection group (Fig. 6b), suggesting that USP28 SUMOylation regulates the transcriptional activity of HIF-1α. In summary, USP28 K99R had higher activity than USP28 WT in stabilizing and activating HIF-1α.

## Discussion

In the present study, we proved that USP28 was SUMOylated under normoxic condition but rapidly deSUMOylated during hypoxia. We further showed that SUMOylation at K99 regulates the deubiquitinating activity of USP28 towards HIF-1α. We also demonstrated that SENP1 directly interacts with and also deSUMOylates USP28. In USP28 knockdown cells, the HIF-1α stabilization activity of SENP1 was largely impeded. Thus, our data revealed a previous unknown SENP1–USP28–HIF-1α positive feedback loop to maximize the accumulation of HIF-1α protein and amplify its downstream effects during hypoxia response (Fig. 6c). In line with these observations, cells with K99R mutant USP28 overexpression exhibited increased HIF-1α protein level and enhanced HIF-1α transcriptional activity.

Previous studies have revealed that expression of USP28 was strongly elevated in human colon and breast carcinomas, and overexpression of USP28 enhanced HIF-1α-dependent angiogenesis and promoted colorectal cancer [4, 17, 18], suggesting that it might play a critical role in tumor cellular pathways. SENP1 has also been shown to be essential for HIF-1α accumulation during hypoxia by directly interacting and deSUMOylating HIF-1α protein [8]. Our data further elucidated that SENP1-mediated USP28 deSUMOylation was indispensable for its deubiquitinating activity towards HIF-1α, indicating SENP1 associated with USP28 to fully activate the HIF-1α pathway. Thus, the results in this manuscript filled a mysterious gap in the accumulation and activation of HIF-1α during hypoxia response. Moreover, it will be interested to further explore whether the activity of other deubiquitination enzymes were also regulated by SUMOylation and deSUMOylation circle, and whether other SENP family members also participated in this regulatory process.

Furthermore, our data have extended the understanding of the mechanisms mediating the accumulation of HIF-1α during hypoxia and introduced a new cross-talk between SUMOylation and ubiquitination in controlling HIF-1α stabilization. The posttranscriptional regulation by SENP1–USP28–HIF-1α axis may constitute a novel cellular adaptive response to allow HIF-1α maximal accumulation in response to hypoxia signals that affect cells survival, angiogenesis, and even tumorgenesis. Thus, further functional works are warranted to further clarify the exact roles of this axis in the board biological processes. Nevertheless, our studies offer the opportunity to shutdown HIF-1α-dependent downstream effects during hypoxia response by targeting SUMO modified USP28, and setup the stage for relevant drug discoveries in future researches.

## Conclusions

The present study found that the deubiquitinase USP28 was SUMOylated at K99 under normoxic condition but rapidly deSUMOylated by SENP1 during hypoxia. USP28 SUMOylation regulates its deubiquitinating activity against HIF-1α. Overexpression of a K99R USP28 mutant in cells significantly increased HIF-1α protein level and enhanced HIF-1α transcriptional activity. These founding reveals a previous unknown SENP1–USP28–HIF-1α positive feedback loop and offers a novel target for cancer therapy.

## Abbreviations

GSK-3: glycogen synthase kinase-3
HIF-1α: hypoxia-inducible transcription factor-1α
HRE: hypoxia response elements
IP: immunoprecipitation
shRNA: small hairpin RNA
SENP: SUMO1/sentrin specific peptidase
USP28: ubiquitin-specific protease 28
VHL: Von Hippel–Lindau
